# Why the Convention on the Rights of the Child must become a guiding framework for the realization of the rights of children affected by tuberculosis

**DOI:** 10.1186/s12914-016-0105-z

**Published:** 2016-12-08

**Authors:** Robindra Basu Roy, Nicola Brandt, Nicolette Moodie, Mitra Motlagh, Kumanan Rasanathan, James A. Seddon, Anne K. Detjen, Beate Kampmann

**Affiliations:** 1 Centre for International Child Health, Department of Paediatrics 2nd Floor, Medical School Building, St Mary’s Campus, Imperial College London, London, W2 1PG UK; 2 Vaccines and Immunity Theme, Medical Research Council (MRC) Unit The Gambia, Fajara, The Gambia; 3 Human Rights Unit, Programme Division, United Nations Children’s Fund (UNICEF), 3 UN Plaza, New York, NY USA; 4 Human Rights Unit, Programme Division, UNICEF, 5-7 Avenue de la Paix 1211, Geneva, Switzerland; 5 Health Section, UNICEF, 3 UN Plaza, New York, NY USA; 6 Childhood TB, UNICEF, 3 UN Plaza, New York, NY USA

**Keywords:** Human Rights, Tuberculosis, Paediatrics/Pediatrics, Human Rights-Based Approach, Stigma, Non-discrimination, Autonomy, Medical ethics, Child health, Child survival

## Abstract

**Background:**

Until recently, paediatric tuberculosis (TB) has been relatively neglected by the broader TB and the maternal and child health communities. Human rights-based approaches to children affected by TB could be powerful; however, awareness and application of such strategies is not widespread.

**Discussion:**

We summarize the current challenges faced by children affected by TB, including: consideration of their family context; the limitations of preventive, diagnostic and treatment options; paucity of paediatric-specific research; failure in implementation of interventions; and stigma. We examine the articles of the Convention on the Rights of the Child (CRC) and relate them to childhood TB. Specifically, we focus on the five core principles of the CRC: children’s inherent right to life and States’ duties towards their survival and development; children’s right to enjoyment of the highest attainable standard of health; non-discrimination; best interests of the child; and respect for the views of the child. We highlight where children’s rights are violated and how a human rights-based approach should be used as a tool to help children affected by TB, particularly in light of the Sustainable Development Goals and their focus on universality and leaving no one behind.

**Summary:**

The article aims to bridge the gap between those providing paediatric TB clinical care and conducting research, and those working in the fields of human rights policy and advocacy to promote a human rights-based approach for children affected by TB based upon the Convention on the Rights of the Child.

## Background

Human rights-based approaches to health can be helpful in improving health outcomes while transforming the underlying or root causes for how disease is distributed (see definition Box 1) [1–7]. But how can we use the framework of human rights, and the Convention on the Rights of the Child (CRC) in particular, to help guarantee the rights of children affected by TB?[8]

The CRC, the most widely ratified human rights treaty in history (Box 2), must become a guiding framework for all of us working to improve the health and situation of children and families affected by TB [9, 10] Here, we take a critical look at the CRC, and other documents unpacking the right of the child to the highest attainable standard of health, and how these norms and key principles (such as non-discrimination, participation, and accountability) can be leveraged, particularly in the context of the 2030 Agenda for Sustainable Development that seeks to realize the rights of all and leave no one behind (Table 1). In support of this ambitious goal, the Global Strategy for Women’s, Children’s and Adolescents’ Health (2016–2030) aims to achieve the highest attainable standard of health for all women, children and adolescents, transform the future and ensure that every newborn, mother and child not only survives, but thrives. (http://www.everywomaneverychild.org/)

**Table 1:** The broader context of Sustainable Development for children affected by TB

The 2030 Agenda for Sustainable Development was adopted by the Member States of the United Nations in September 2015. This agenda outlines a vision of integrated social, economic and environmental development for all people in all countries in the next 15 years, underpinned by the human rights principles of universality, non-discrimination, participation and accountability. The agenda contains the 17 Sustainable Development Goals (SDGs) and 169 accompanying targets, which succeed the Millennium Development Goals, but this time aiming to ensure that “no one is left behind”. There is a specific goal 3 on health, but targets across the agenda are fundamental to realizing the right to health for all. The Global Strategy for Women’s, Children’s and Adolescents’ Health, launched alongside the 2030 Agenda, highlights 17 key SDG targets across the agenda that are key for health.		
SURVIVE:	THRIVE:	TRANSFORM:
*End preventable deaths*	*Ensure health and well-being*	*Expand enabling environments*
- Reduce global maternal mortality to less than 70 per 100,000 live births - Reduce newborn mortality to at least as low as 12 per 1000 live births in every country - Reduce under-5 mortality to at least as low as 25 per 1000 live births in every country - End epidemics of HIV, tuberculosis, malaria, neglected tropical diseases and other communicable diseases - Reduce by one third premature mortality from non-communicable diseases and promote mental health and well-being	- End all forms of malnutrition, and address the nutritional needs of adolescent girls, pregnant and lactating women and children - Ensure universal access to sexual and reproductive health-care services (including for family planning) and rights - Ensure that all girls and boys have access to good quality early childhood development - Substantially reduce pollution-related deaths and illnesses - Achieve universal health coverage including financial risk protection and access to quality essential services, medicines and vaccines	- Eradicate extreme poverty - Ensure that all girls and boys complete free, equitable and good quality primary and secondary education - Eliminate all harmful practices and all discrimination and violence against women and girls - Achieve universal and equitable access to safe and affordable drinking water and to adequate sanitation and hygiene - Enhance scientific research, upgrade technological capabilities and encourage innovation - Provide legal identity for all, including birth registration - Enhance the global partnership for sustainable development

We start with the scenario of a family affected by TB, incorporating realities from clinical cases, to draw out the key issues pertaining to childhood TB and the systems failing to address it. We then look at the CRC and relate its framework to paediatric TB, starting with the right to life and the right to the highest attainable standard of health (the ‘right to health’), and States’ duties towards these rights of children, and their survival and development. We go on to focus on the other core principles of the CRC: non-discrimination; best interests of the child; and respect for the views of the child [11]. We conclude with a call-to-arms for policy-makers, researchers, clinicians, society, advocacy groups, families, and children and adolescents themselves. Human rights are increasingly part of an evolving dialogue in global health and development [12]. We seek to bridge the gap between those involved in clinical care and research in paediatric TB and those working in the fields of human rights policy and advocacy. Bridging this gap and applying human rights-based approaches to children affected by TB is key if by 2030 we wish to deliver on our promise to place children and adolescents at the heart of implementing the Sustainable Development Goals, and end TB [13].

### Tuberculosis – a family disease

We start with vignettes of two families affected by TB – one family where the health and social care systems are struggling, and another family where the health and social infrastructure are able to operate and respond more effectively (Table 2). The vignettes illustrate numerous distinctive features of paediatric TB, including the even more complex threat of multidrug-resistant (MDR)-TB. TB can occur almost anywhere - in both developed and less developed countries. The scenario of the first family exemplifies failures that ultimately deny children their rights to life and health: failures in screening contacts of TB patients; failures to provide preventive therapy; diagnostic delays; poor access to health; lack of appreciation that paediatric TB is not the same as adult TB; treatment failures; and systems failures. Although this particular scenario involves MDR-TB, most of the failures equally apply to children suffering from drug-susceptible TB. In contrast, the second family benefits from an integrated approach between health, education, and social care, highlighting the interdependence and indivisibility of children’s rights.

**Table 2:** Illustrative case vignettes of families affected by TB, highlighting the inequality in existing health provision. Key issues raised by the vignettes are summarized on the right

Tuberculosis – a family disease across the world		
Struggling health and social care system	Effective health and social care system	Key lessons illustrated
Six-year-old Jamil lives with his mother and 2-year-old sister, Zahra, in a rural village in a low income country. Jamil has been coughing for weeks. His mother has taken him to the traditional healer, but he worsens. She takes him to the nearest clinic where the healthcare worker gives antibiotics with no improvement. Eventually he is referred to the nearest hospital where an x-ray is taken and he is diagnosed with pneumonia and further antibiotics are prescribed. Repeated sputum examinations show no signs of TB but given the lack of response he is started on the standard four-drug TB regimen. He continues to deteriorate and so the health care workers explore Jamil’s case further. They realize that Jamil’s father had similar symptoms following his release from prison many months ago and was eventually diagnosed with multidrug-resistant TB and is an inpatient at the national sanatorium. Jamil is therefore referred three hours away to the national children’s hospital, where he starts MDR-TB treatment with daily injections and tablets. His mother, already struggling with an absent father does not have the means to visit him. Jamil will remain an inpatient for 8 months, separated from his family. His schooling stops and he gradually loses his hearing due to the medication he is receiving. Shortly after Jamil is admitted to the national children’s hospital, his younger sister, Zahra, becomes lethargic and spikes fevers. One day, his mother is unable to wake her up and she is taken by cart to the nearest hospital. The doctors suspect TB meningitis and start her on treatment, but she dies two days later.	Six-year-old Jamil lives with his mother and 2-year-old sister, Zahra, in social housing in a large city of a high income country. Public health officials visit the family as part of a contact tracing program because their father was recently diagnosed with multidrug-resistant TB following his release from prison several months ago. Jamil’s youngest sister Zahra is 2 years old. Although she appears healthy and her investigations are normal, given her young age and close contact with a known MDR-TB case, her team of doctors start her on medication to prevent her from developing TB disease. Jamil has no symptoms yet, but the public health team request a chest radiograph and other tests, the results of which suggest he has early TB disease. Given his father is known to have MDR-TB, he is admitted to the regional children’s hospital where he undergoes paediatric-specific investigations for TB and he is treated with injections and tablets. He receives education from the hospital teaching team whilst an inpatient. His case is discussed at the multidisciplinary meeting to help his mother access benefits enabling her to visit him in hospital. Once his treatment regime has been stabilized, an arrangement is made between the hospital and the school so that he returns to live at home and attends the hospital daily after school. Following questions raised by Jamil and his family, the public health authorities liaise with the school to reassure and educate that there is no risk of transmission to others as he is on treatment and not coughing. In view of the potential side effects of the drugs that Jamil is taking, his hearing is tested regularly. After 2 months of treatment, there are early signs that his hearing is affected so his medication regimen is changed before he experiences hearing loss that might compromise his ability to communicate. As his disease was identified early, he is treated for a shorter duration of therapy then is required for adults with extensive disease.	• TB disproportionately affects marginalized populations across the world – e.g. those living in poverty, difficult access to healthcare, migrants and refugees. • Effective public health mechanisms and infection control measures are necessary to identify linked cases and prevent further transmission. • Contact tracing can lead to identification of contacts eligible for therapy to prevent TB disease developing. • Contact tracing can lead to early detection and treatment of paediatric TB cases. • Understanding the differences between adult and paediatric TB is key to diagnosis and treatment initiation. • Continued education is possible and requires coordination of health, education, and social sectors. • Even when separation of children and their families during treatment is necessary, the impact can be minimized and the duration limited to the absolute minimum. • Children can and should be involved in and understand their own care and be communicated with in an age-appropriate manner. • Education of communities and increased awareness around TB will reduce stigma, diagnostic delays and improve access and uptake of TB services. • Appropriate provision of care to children affected by TB can prevent disability and death.

### Paediatric tuberculosis – where are we now?

It is estimated that 2–3 billion people worldwide are infected with *Mycobacterium tuberculosis* (the organism that causes TB) [14]. For the majority, the organism will live in the host without causing illness, contained in a latent state by their functioning immune system. However, from this vast reservoir, millions each year progress to a disease state in which the organism overcomes the immune system and TB disease develops with characteristic features of fever, weight loss, night sweats and cough [15]. Factors such as under-nutrition, poor housing conditions, and limited access to healthcare form an inexorable link to susceptibility and transmission of TB amongst marginalized populations [16]. Those at the highest risk of progression from infection to disease are people with an impaired immune system (such as people with HIV, diabetes, or malnutrition) and young children, especially those under the age of five [17]. Following exposure to an infectious case of TB in the household, about half of children will become infected [18]. The children in our vignettes were likely infected in their home by their father. In cases of drug-susceptible TB, preventive therapy with a single drug given to healthy child contacts can prevent the progression from infection to disease. Yet, the routine screening of contacts and provision of preventive therapy to those at high risk is rarely implemented in TB high burden settings [19].

As well as having a high risk of progressing from infection to disease, children, especially those under 5 years old, have a higher risk than adults of developing severe clinical presentations of TB beyond the lungs. These include meningitis (as shown in our vignette), and disseminated ‘miliary’ TB, which are associated with a higher risk of dying and disability [20]. Confirming a diagnosis of TB in children is a challenge as children can become ill with relatively few bacteria, meaning that microbiological tests may miss the majority of cases [21]. Most cases of paediatric TB are therefore diagnosed clinically but as the symptoms, and clinical and radiological signs are non-specific, under- and over-diagnosis occur [22].

Children affected by TB can suffer from stigma, isolation and poor access to education – either whilst attending health centres regularly for Direct Observed Therapy, or whilst admitted for prolonged inpatient treatment, or through stigma preventing school attendance [23]. As TB is a family disease, even children that do not develop TB can suffer: 10 million children were orphaned in 2010 by the death of a parent from TB [24]. The link between vulnerable and poor populations bearing the brunt of the impact of TB is well-established, with economic, geographic, gender, sociocultural and health systems barriers providing mechanisms for a vicious cycle of perpetuation and exclusion of already marginalized populations [16].

Public health programs and international agencies have focused policy and statistics on adults with microbiologically confirmed TB as the primary contributors to transmission in the community. Estimates of the disease burden of tuberculosis in children have only been included in the annual WHO Global Report on TB since 2012 [25] The latest figures estimate that each year one million children develop TB across the world, with 136,000 children dying from the disease [14] Global inequalities are highlighted by the African Region having the most severe burden relative to population in 2014, with 281 cases for every 100 000 people, more than double the global average of 133 [14]. Worryingly, we are only detecting 36% of the 1 million children with TB worldwide. Much more needs to be done to identify children at risk for TB and provide them access to quality prevention, diagnosis and care. The entry point of children with TB into the health system is usually community and primary health care services. Therefore, paediatric TB needs to be better integrated into broader maternal and child health programs.

Diagnosis and treatment of MDR-TB (caused by *M. tuberculosis* resistant to two first-line drugs: rifampin and isoniazid) in children is challenging, and although there have been treatment guidelines published in recent years, these are fundamentally limited by the paucity of pharmacodynamic and pharmacokinetic data in children, and the lack of suitable formulations of the drugs for young patients [26]. Treatment is long and often toxic, children suffer from side effects such as nausea, vomiting, diarrhea, irreversible hearing loss (such as Jamil) and abnormal thyroid function [27]. In low resource settings, capacity to diagnose and manage children with MDR-TB is often restricted to national level tertiary care settings or specialized MDR-TB hospitals. As a result, children are sent far away for months of inpatient treatment.

We are not the first to consider paediatric TB a “neglected disease” [28]. In recent years there has been increasing emphasis on the need for action, with calls for a stronger advocacy approach [29] The Sustainable Development Goals and WHO End TB strategy are now aiming to end TB and for zero childhood TB deaths, so rapid and dramatic progress is required to make this a reality [30] The TB community has historically not embraced a human rights-based or child-focused approach, nor has it strongly engaged in advocacy. Lessons can be learned from other communities such as HIV/AIDS, which is far ahead of TB on adopting human rights-based approaches. The CRC can be a guiding framework for the TB community on all actions that have to be taken, not only in regard to the care that children affected by TB receive, but also more broadly in terms of legislation, resource allocation or capacity building, to ensure that everything possible has been done to guarantee the rights of all children affected by TB and their families, and thus address the failures shown in the vignette. It is a tool that those affected by, and those advocating for and managing TB, need to learn how to use. Drawing on aspects of the clinical scenarios and the features of childhood TB presented here, we now go on to consider how the CRC can and should be used as a guiding framework to improve the situation of children affected by TB.

The CRC, which the United Nations General Assembly adopted over 26 years ago, was and continues to be a revolutionary document (Box 2). Outlining universal principles and standards for the promotion and protection of the rights of the child, the Convention explicitly recognizes children as social actors and active holders of their own rights, rather than as objects of charity. With a view to providing States parties with guidance on how to deliver on their obligations regarding the child’s right to health, the Committee on the Rights of the Child (hereafter referred to as The Committee) developed a General Comment (i.e., an authoritative interpretation) on the right of the child to the enjoyment of the highest attainable standard of health to further elaborate on Article 24 of the Convention [31] This General Comment provides invaluable insight on the definition of this right and is addressed to a wide range of stakeholders from public health professionals, to policymakers, as well as the private sector. It provides helpful indications of key considerations for the provision of care to children affected by TB.

### Back to basics – what is the definition of the child?

As this article aims to identify how the CRC framework should be used to support the realization of the rights of children affected by TB, we must start by taking a closer look at the definitions it provides. Article 1 (Table 3) defines a child as “every human being below the age of 18 years unless under the law applicable to the child, majority is attained earlier” [8]. This definition is the result of important negotiations as some States had argued for a lower age limit, since it is linked not only to the concept of children as a subject of rights to be progressively exercised in accordance with their evolving capacities, but also to the obligation of States to provide special protection.

**Table 3:** Key excerpts from the Convention on the Rights of the Child related to children affected by tuberculosis

CRC Reference	Key text
Preamble	The United Nations has proclaimed that childhood is entitled to special care and assistance…Recognizing that the child, for the full and harmonious development of his or her personality, should grow up in a family environment, in an atmosphere of happiness, love and understanding…Recognizing that, in all countries in the world, there are children living in exceptionally difficult conditions, and that such children need special consideration; Recognizing the importance of international cooperation for improving the living conditions of children in every country, in particular in the developing countries
Article 1	For the purposes of the present Convention, a child means every human being below the age of 18 years unless under the law applicable to the child, majority is attained earlier.
Article 2	1: States Parties shall respect and ensure the rights set forth in the present Convention to each child within their jurisdiction without discrimination of any kind, irrespective of the child’s or his or her parent’s or legal guardian’s race, colour, sex, language, religion, political or other opinion, national, ethnic or social origin, property, disability, birth or other status. 2. States Parties shall take all appropriate measures to ensure that the child is protected against all forms of discrimination or punishment on the basis of the status, activities, expressed opinions, or beliefs of the child’s parents, legal guardians, or family members.”
Article 3	1. In all actions concerning children, whether undertaken by public or private social welfare institutions, courts of law, administrative authorities or legislative bodies, the best interests of the child shall be a primary consideration 3. States Parties shall ensure that the institutions, services and facilities responsible for the care or protection of children shall conform with the standards established by competent authorities, particularly in the areas of safety, health, in the number and suitability of their staff, as well as competent supervision
Article 6	1. States Parties recognize that every child has the inherent right to life. 2. States Parties shall ensure to the maximum extent possible the survival and development of the child.
Article 9	1. States Parties shall ensure that a child shall not be separated from his or her parents against their will, except when competent authorities subject to judicial review determine, in accordance with applicable law and procedures, that such separation is necessary for the best interests of the child.”
Article 12	The child who is capable of forming his or her own views the right to express those views freely in all matters affecting the child, the views of the child being given due weight in accordance with the age and maturity of the child.”
Article 22	1. States Parties shall take appropriate measures to ensure that a child who is seeking refugee status or who is considered a refugee in accordance with applicable international or domestic law and procedures shall, whether unaccompanied or accompanied by his or her parents or by any other person, receive appropriate protection and humanitarian assistance in the enjoyment of applicable rights set forth in the present Convention and in other international human rights or humanitarian instruments to which the said States are Parties.”
Article 23	1. States Parties recognize that a mentally or physically disabled child should enjoy a full and decent life, in conditions which ensure dignity, promote self-reliance and facilitate the child’s active participation in the community
Article 24	1. States Parties recognize the right of the child to the enjoyment of the highest attainable standard of health and to facilities for the treatment of illness and rehabilitation of health. States Parties shall strive to ensure that no child is deprived of his or her right of access to such health care services.”

It is important to note the difference between the definition provided by the CRC and that used by WHO in the annual Global Tuberculosis Report that provides a comprehensive assessment on the present situation of TB across the world, and is used as the benchmark against which to judge progress and ongoing challenges in combatting TB. The report defines children as people below the age of 15 years in line with existing criteria for notification of TB cases to public health authorities in member countries [14]. Reporting age strata from 0–4, 5–14 and then 15–24 years results in the inability to identify and present the needs and challenges of adolescents affected by TB. Adolescence is associated with an increasing risk of pulmonary tuberculosis and accompanying transmission to others [17]. By statistically lumping adolescents with young adults, little attention is paid to the specific health, educational and emotional needs in this critical phase of life.

### A right to life and the highest attainable standard of health

The CRC clearly states that children have both an inherent right to life in Article 6 (Table 3) and to the highest attainable standard of health, enshrined in Article 24 (Table 3). While the latter right to health does not equate to the right to good health, States parties realize the right to health by ensuring that the necessary systems and values are in place for all children within their jurisdiction. It gives rise to legally binding obligations and also requires States to establish adequate monitoring and accountability mechanisms. Building on the earlier General Comment of the Committee on Economic, Social and Cultural Rights which helped unpack the concept of a human rights-based approach to health, [32] the Committee developed a General Comment 15, which states that:

“The notion of “the highest attainable standard of health” takes into account both the child’s biological, social, cultural and economic preconditions and the State’s available resources, supplemented by resources made available by other sources, including non-governmental organizations, the international community and the private sector.” [33]

The Committee goes further to point out that:

“most mortality, morbidity and disabilities among children could be prevented if there were political commitment and sufficient allocation of resources directed towards the application of available knowledge and technologies for prevention, treatment and care.” [31]

Explicitly, preventing disease, disability and death is within the power of the global community and the Committee therefore states the requirement “to ensure the availability, accessibility, affordability, acceptability and quality of facilities, goods and services related to health, as well as to address its underlying determinants, such as poverty, poor education and lack of access to other social services” [6].

Children such as Zahra from our vignette who develop TB meningitis have mortality of 1 in 5 with more than half of survivors left with chronic neurological and development impairments [34]. Where children have disabilities resulting from TB, States parties must ensure care that is centred on promoting and maximizing the child’s abilities. Children with disabilities in low- and middle-income countries are amongst the most vulnerable and neglected by health and education systems [33]. Article 23 of the CRC outlines the right of children with disabilities to a “full and decent life in conditions which ensure dignity, promote self-reliance and facilitate the child’s active participation in the community”, requiring that States parties ensure the special care and assistance so that the child – in this case children with disabilities resulting from TB - can achieve the “fullest possible social integration and individual development, including his or her spiritual or cultural development.”

TB is a preventable disease, and a number of measures are known to be effective in preventing TB in children as shown in the vignettes. These include household contact tracing, preventive therapy and infection control [35]. Why is it that these successful interventions are the cornerstone of TB programs in high income, low TB burden settings but rarely implemented as part of routine care in TB-endemic countries? Part of it might be the lack of sufficiently strong links between TB services and community-based providers that are ideally placed to perform contact screening. But it is also a lack of resources. The latest figures from the WHO suggest that there is a $1.4 billion funding gap to implement existing interventions [14]. By failing to implement such policies, the global community is guilty by inaction of preventing childhood morbidity, mortality and disability attributable to TB.

#### Where is the evidence? Where are the children in the studies?

A further recurring theme is the extrapolation of research conducted on adults to the policies, drugs, doses and formulations with which we treat children affected by TB.

The Committee has emphasized that implementation of article 24 “…must be shaped by evidence-based public health standards and best practices” [31]. It has also noted ensuring quality requires, *inter alia,* that treatments, interventions and medicines are based on the best available evidence [33].

It is therefore important for States to mobilize funding for such research and provide a regulatory framework that ensures children are included in current and future trials to provide the epidemiological data, the study results and the evidence base to determine the standards and best practices that should then be implemented. An excellent example of the States’ key role in this area is European regulation instituted in 2006 that mandated Paediatric Investigation Plans for the development and authorization of medicinal products for the paediatric population subsets [36]. The need to improve data reporting and to address paediatric-specific research gaps in epidemiology, basic science, diagnostics, therapeutics, vaccines and operational research are highlighted as essential in the goal of zero childhood TB deaths [30].

#### Drug treatment in children

The Committee has explicitly stated that quality of services requires, *inter alia,* that drugs are child-specific (when necessary) [31]. Merely adjusting adult treatment practices for children can result in over- or under-dosing, an assumed length of treatment that may or may not be optimal for children, and toxicities that could potentially be avoided. Children metabolize drugs more rapidly than adults and a higher relative dosage is required in children to attain the same serum concentration as is achieved in adults. Until a recent revision by the WHO, the same dosages and durations of treatment had been given to children as advised for adults [37]. There are significant and irreversible side effects with some MDR-TB drugs such as the deafness experienced by Jamil in our vignette. Access to both these old toxic drugs and newly licensed drugs for MDR-TB is poor, thereby depriving children with MDR-TB of their right to the highest attainable standard of health [38–41]. There is a precedent for seeking legal remedies to ensure paediatric drug TB availability, with the Indian National Human Rights Commission raising the issue of drug supply, and specifically paediatric formulations with the Government [42]. Traditionally, children were treated with adult drug formulations and to provide paediatric dosing, tablets had to be cut, broken or ground, resulting in inaccuracies, with implications for both efficacy and toxicity. In December 2015, the first Fixed Dose Combinations designed for children finally became available, which represents a landmark moment in provision of suitable care to children affected by TB [43]. Studies are underway to see if children can be treated with shorter durations of therapy than the current 6 month regimen as they typically have fewer bacteria than adults with TB [44].

#### Where, how, and by whom, should care be provided to children affected by TB?

“States should ensure an appropriately trained workforce of sufficient size to support health services for all children. Adequate regulation, supervision, remuneration and conditions of service are also required, including for community health workers. Capacity development activities should ensure that service providers work in a child-sensitive manner and do not deny children any services to which they are entitled by law. Accountability mechanisms should be incorporated to ensure that quality assurance standards are maintained.” [31]

This call by the Committee for ‘child-sensitive’ care echoes the universal refrain of paediatricians around the world that children are not small adults. As discussed, children affected by TB range from neonates to adolescents, each needing age and developmentally appropriate clinical skills, interpretation of investigations, communication techniques, consideration of their educational needs and so forth.

Children die of TB because health professionals are not trained to recognize and diagnose paediatric TB, a failure to meet Article 3.3 (Table 3). Some skillsets and knowledge need to be considered universal and available through training for primary health care workers. Increasing global access to the internet brings huge opportunities for training materials to have widespread impact, such as the Childhood TB Learning Portal [45]. A continuum of care is needed – which can appropriately manage children of all ages with strong recognition and referral systems for those children at risk of TB starting with primary health care at the community level. There is a distinct lack of health care infrastructure designed with children in mind across the world, and particularly in TB-endemic countries [46]. Especially when hospitalization is prolonged, or where access to school is limited in infectious phases of disease or due to stigma, continuing education for young people affected by TB is crucial. Too often, children are an afterthought when guidance is formulated. Article 3.3 of the CRC can therefore be used as leverage to ensure that it is incumbent upon governments and health services to implement best practice as recommended by the WHO, and to ensure that those caring for children affected by TB are appropriately trained and in sufficient numbers to cope with the health needs.

### Non-discrimination

The CRC provides a strong normative framework for all children everywhere to get the best start in life, to survive and thrive. The Committee also reaffirmed on several occasions the universality, indivisibility and interdependence of the rights contained in the Convention. The legally binding nature of the Convention requires State parties to undertake all appropriate legislative, administrative, and other measures (including by developing national strategies rooted in the Convention, ensuring adequate financing for children, or by setting up an independent human rights institution) for the implementation of the rights recognized in the Convention. The Committee also reaffirmed the obligation of States to facilitate the implementation of children’s right to health by all actors.

This obligation takes many forms. With one million cases of childhood TB a year, it is important to see if the right environment is in place to address the various facets of this disease. As discussed, children and adolescents encompass a varied group who will face different challenges in the context of TB – from the neonate being breastfed by a mother with TB; to the teenager unable to access education and stigmatized because of this disease. As the State’s responsibility is to *every* child, our collective actions against TB must protect the newborn, the infant, the pre-school child (Zahra from our vignette), the school-age child (Jamil from our vignette) and the adolescent, with a particular focus on those children who are living in poverty, or are in other ways marginalized, economically and/or socially excluded. For instance, TB rates have been shown to be several fold higher in indigenous populations in Brazil, Canada, Greenland and Canada, although paediatric specific data is (again) limited [16, 47, 48]. Article 2 (Table 3) of the CRC makes explicit the principle of non-discrimination for the overall implementation of the Convention.

The grounds for discrimination mentioned in the Convention mirror those stated in the International Covenants on Civil and Political Rights and on Economic, Social and Cultural Rights, with the addition of ethnic origin and disability. However, due to the reference in Article 2 to “other status”, this is not an exhaustive list. The Committee also identified additional grounds for discrimination in paragraph 8 of General Comment 15, which refers to sexual orientation, gender identity and health status, for example HIV status and mental health [31] Discrimination can be overlapping, or intersectional, with the most marginalized children experience discrimination due to multiple factors. The application of the principle of non-discrimination is thus key to addressing inequalities in the impact of TB.

### Underlying determinants of children’s health

#### Sex, Gender and childhood TB

Males and females are affected in different ways by TB. There is a male predominance in case notifications, and differences between males and females in prevalence of infection, rate of progression from infection to disease, incidence of clinical disease, and mortality due to TB [49]. Biological and sociocultural effects contribute to such differences, including hormonal and pubertal effects on immunity, patterns of exposure, access to care and diagnosis, support for treatment completion, and the skewed sex distribution of HIV/AIDS in different regions of the world [50]. General Comment 15 (paragraph 9) also emphasizes the negative impact of gender-based discrimination on health outcomes, and the need to consider the impact of social norms and values on the health of girls and boys [31].

Furthermore, Article 24.2(d) of the CRC and General Comment 15 paragraph 18 recognize that mothers’ health and rights are fundamental to children’s health. There is increased risk of perinatal mortality and prematurity when TB occurs during pregnancy [51] Children of a mother with TB can be highly exposed through breastfeeding, bed sharing, and the high degree of interaction, contributing to why young children represent the most vulnerable of age groups [19]. The Convention clearly stipulates that the State’s obligation to fulfill children’s right to health therefore also requires appropriate pre-natal and post-natal health care for mothers. Separation of breastfeeding infants from their mothers affected by TB is often unnecessary but still very commonly done [35].

#### Migration and childhood TB

Data collected by the United Nations Department of Economic and Social Affairs (UN/DESA) and UNICEF show that there are over 35 million international migrants under the age of 20 [52]. The reasons behind families migrating vary from fleeing violence, discrimination or conflict in their countries of origin, to seeking better economic and social opportunities in destination countries. In general, and particularly in the current era of unprecedented movements of large numbers of refugees and migrants to Europe away from conflict and instability in the Middle East, States and the wider international community have a duty of care to the children involved, as stated in Article 22 of the Convention (Table 3) [53]. In fact, Article 2 explicitly states that States parties have an obligation towards all children *within their jurisdiction*, i.e. not just towards their citizens.

Specifically, the Committee has indicated that States should ensure that all children, whether citizens or not, should have the same access to economic, social and cultural rights and to basic services, regardless of their or their parent’s migration status, and should make this explicit in legislation [54].

Human movements on such a large scale raise acute issues at the time of arrival in the host or transit countries, including the screening of large numbers of physically and emotionally exhausted children and their families, some of whom may have active TB disease. Longer term implications include the progression of individuals from asymptomatic TB infection to TB disease, potentially years later in the final destination country where opportunities for contact tracing, preventive treatment, and the identification of further cases may have been lost [55]. Refugee and migrant children face particular barriers to realizing their right to health, with reduced access to, and utilization of, both preventive and general health care services [56].

#### Tuberculosis and poverty

The link between TB and poverty has long been recognized and touched upon already in this article [16]. Children affected include those living in low-income countries but also poor families in high-income settings. Poor families such as those in our vignette often have less access to healthcare as well as less ability to pay for it. As Archbishop Desmond Tutu said “TB is the child of poverty – and also its parent and provider” [57]. The poor are at the greatest risk of being exposed to TB, have the highest prevalence of disease, have lower treatment completion rates, and suffer the highest mortality [16]. Vulnerable populations such as migrants, refugees, the homeless, prisoners, and people living with HIV are disproportionately affected. Their plight is made worse by loss of earning potential, loss of educational opportunities, by the costs associated with accessing healthcare because of having TB, and finally through stigma, highlighting the interdependence and indivisibility of rights [58]. The End TB strategy explicitly incorporates that no family affected by TB should have catastrophic costs related to TB [13].

Recognizing that poverty is also a key determinant of health for children, the Committee states, for example, that:

“Barriers to children’s access to health services, including financial, institutional and cultural barriers, should be identified and eliminated. Universal free birth registration is a prerequisite and social protection interventions, including social security such as child grants or subsidies, cash transfers and paid parental leave, should be implemented and seen as complementary investments…Lack of ability to pay for services, supplies or medicines should not result in the denial of access. The Committee calls on States to abolish user fees and implement health-financing systems that do not discriminate against women and children on the basis of their inability to pay. Risk-pooling mechanisms such as tax and insurance should be implemented on the basis of equitable, means-based contributions” [31]

Within marginalized and vulnerable communities such as those mentioned above, children represent the most vulnerable sub-population. Without a universal, pro-poor, equitable approach, and without addressing the underlying determinants of TB, as well as structural changes in governance and in the knowledge, attitudes and practices of communities, little progress will be made [59]. History has taught us that, even without a single drug against *M. tuberculosis*, simply raising living standards can cause a steady decline in rates of TB [60].

### Best interests of the child

Article 3.1 (Table 3) places an obligation on health workers and hospital administrators to assess the best interests of the individual child in the management of their disease. It also places an obligation on States to make the best interests of children a central consideration in budget allocation and policy development [6]. The Committee has emphasized that the best interests of the child, being “a primary consideration”, cannot be considered at the same level as other potentially competing considerations in decisions affecting children [61]. At the same time, the concept is flexible and adaptable and should be adjusted and defined according to the specific situation of the child, taking into consideration their personal context, situation and needs.

How does this relate to TB? One aspect is the impact of TB on the entire family, and the need to consider and involve children, parents and siblings in the management of their disease. Where should children be provided with their care? If a child is admitted to hospital, how does the family balance its responsibilities to the unwell child on the ward and the siblings left at home? Potential conflicts have to be resolved on a case-by-case basis, carefully balancing the interests of all parties and finding a suitable compromise. In this analysis, greater weight must be given to the best interests of the child or children concerned. An important factor in weighing the competing rights and determining the best interests, is the opinion of the child or children themselves, bearing in mind their evolving capacities.

The recurring theme here is that of TB as a disease of families as seen in our clinical examples. Children are usually infected by adults with pulmonary TB. Particularly when considering MDR-TB, there are tensions between maintaining the integrity of the family unit, providing medical care to those who are unwell (necessitating hospitalization and intravenous/intramuscular treatment) and preventing transmission of this particularly dangerous form of TB to other vulnerable family members and within society as a whole. WHO infection control guidance suggests that where there are MDR-TB cases in the household “Children below 5 years of age should spend as little time as possible in the same living spaces as culture-positive MDR-TB patients” [62]. The concrete impact of this is separation of children from their parents. Given the delays caused by both patients as well as the health system, the majority of exposure and infection occurs well before a diagnosis is made in the index case and treatment is initiated, so improving access to diagnostics and a rapid pathway to appropriate treatment is key to prevention of paediatric infection and subsequent TB, and to keeping families together.

Article 9, read together with Article 3.1. (Table 3), highlights the balance required between the needs of the parents, the rights of the child, and the interests of society in terms of preventing spread of TB and MDR-TB. Separation can involve a child, a parent, or both being admitted to hospital for prolonged periods, as in the vignettes. The CRC in articles 3.1 and 9 places a firm obligation on States to only allow such separation if it is either necessary in the best interests of the child, or if those best interests are found, on a careful analysis, to be outweighed by the competing rights of others. Where such separation does occur, Article 9.3 requires States to “respect the right of the child who is separated from one or both parents to maintain personal relations and direct contact with both parents on a regular basis, except if it is contrary to the child’s best interests”. In this way the CRC mandates us to find better solutions, including shifting the care of patients away from centralized institutions to community-based care, which is possible even for MDR-TB [63].

### Respect for the views of the child

One of the most revolutionary aspects of the CRC, particularly when it was adopted, is that it recognizes that children have the right to participate in society and express themselves. A wide range of provisions in the CRC guarantee the child’s right to have its views heard and respected in matters concerning them – according to their age and maturity. One of the key articles in this respect is Article 12 (Table 3), which has significant implications for the way in which healthcare treatment and health services are provided [64].

Where and how can we then hear the voices of children affected by TB? Are these children meaningfully and actively involved in decision-making regarding their own healthcare? Too often these children are vulnerable, in marginalized communities, hampered by faltering education and lack of opportunity. They lack access to the ear of those in power or with the ability to help. Researchers and policy makers have called for an urgent need for advocacy by, and for, children affected by TB [29]. The Treatment Action Group has initiated a Global TB Community Advisory Board to liaise with policy makers and drug manufacturers, but still the voice of the children themselves is largely absent [65]. There have been notable efforts and improvements in the visibility of childhood TB on the global health radar in recent years [30]. Although data on children affected by TB is now reported routinely, and they are included in the broader End TB post-2015 strategy, [13] lobbying and advocacy still lags far behind the HIV community.

Linked to the voice of the child is the issue of confidentiality – particularly regarding HIV status and the right to discuss their own health in confidence without parental consent when in the child’s best interests. Again, rarely does the staffing or infrastructure in TB facilities enable this. Furthermore, what questions do children affected by TB want answers to? Although child-friendly educational materials about TB are in development, the gap between children affected by HIV is again a stark one.

Beyond the way in which healthcare treatment and health services are provided to children affected by TB, these children must themselves also be meaningfully engaged in bringing accountability for international child rights commitments, including their right to health, closer to the ground. To this end, and with a particular view to the 2030 Agenda, we must work to secure greater access to information for children affected by TB, as well as opportunities to hold governments accountable for their health right obligations, including through people-led, bottom-up accountability strategies.

### Integration of health, social, and legal perspectives

#### Do TB health workers and healthcare systems think about freedoms and entitlements?

According to the Committee, “Children’s right to health contains a set of freedoms and entitlements. The freedoms, which are of increasing importance in accordance with growing capacity and maturity, include the right to control one’s health and body, including sexual and reproductive freedom to make responsible choices. The entitlements include access to a range of facilities, goods, services and conditions that provide equality of opportunity for every child to enjoy the highest attainable standard of health.” [31]

Notions of freedoms and entitlements are rarely part of a medical or nursing school curriculum. Too often the emphasis is on the child as the dependent, the member of a family, with not enough thought for their freedoms within that social context, or for the fact that they hold defined rights in and of themselves. As the CRC clarified over 26 years ago, children are not simply recipients of care by healthcare workers and administered by their families, but instead are legitimate partners in their treatment. The need for age and developmentally-appropriate communication is key to engaging children and young people in their own health. This will allow them to be meaningfully engaged in decision-making concerning their own healthcare, provide them with access to confidential medical counselling, and consent to treatment [64].

#### Health as a silo – indivisibility and interdependence of children’s rights

The Committee has noted that, “Not only is children’s right to health important in and of itself, but also the realization of the right to health is indispensable for the enjoyment of all the other rights in the Convention. Moreover, achieving children’s right to health is dependent on the realization of many other rights.” [31]

**Fig. 1:**
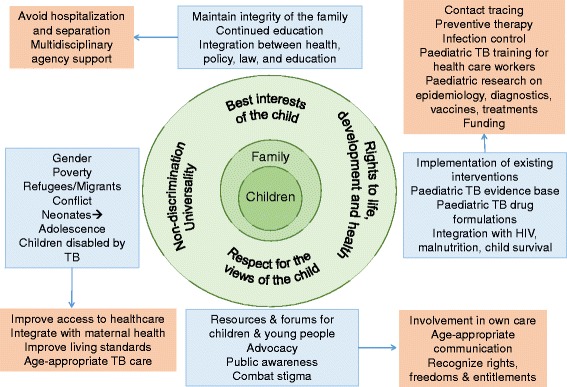
Schematic of the key concepts from the United Nations Convention on the Rights of the Child (CRC) as applied to paediatric TB with the child and family at the center. Adjacent to each concept are themes of relevance to paediatric TB linked to action points for the benefits of children affected by TB. The CRC, as a widely ratified binding legal instrument, opens up avenues to ensure that States parties protect the rights of children affected by TB. States must ensure the justiciability of the right to health and access by children and their families to remedies in case of violation through to civil, criminal or administrative proceedings. Other independent, transparent, and accessible accountability mechanisms such as democratically elected local health councils, patients’ committees, health commissioners, and national human-rights institutions are also key to protection and realization of the rights of children affected by TB

Too often professionals are preoccupied with “their” piece of the puzzle and dialogue, coordination, and appreciation of the work done in fields of education, social work, public health and policy could be improved. It is fundamental that the child should be placed at the centre of the care, and all agencies and actions should be operating in their best interests (Fig. 1). Frequently, vertical programs are implemented focused on particular health priorities such as malnutrition, child health and survival, HIV, and TB, with limited interaction among them, or with primary health care, education, and housing. As a result, there is mis-diagnosis and under-diagnosis, financial and opportunity costs associated with accessing healthcare in multiple locations, and loss to follow-up. Malnutrition is a particularly pertinent example, as malnutrition predisposes to TB, while TB in turn leads to malnutrition, and both are connected to poverty, contributing yet again to the perpetuation of inequity and marginalization [66].

## Conclusion - Finding inspiration and taking action

In the Preamble to the CRC (Table 3), the unique nature of childhood and our accompanying responsibility to nurture and protect it is beautifully expressed. The Convention helps us see children affected by TB as rights holders, and inspires us to incorporate the principles and standards of the CRC for the benefit of our patients and their families.

In real terms, what actions can we take using human rights-based approaches (Fig. 1)? The right to health goes beyond adding power to TB advocacy campaigns, it also adds specific obligations to States. An important first step is ensuring that the right to health is effectively reflected in the State’s legal framework (Constitution, bill of rights, or other statute). This provides important ground not only for more effective national financing but also for the development of other important tools such as national health plans. The CRC, as a widely ratified binding legal instrument also opens up avenues to hold States parties to account to ensure that they protect the rights of children affected by TB. It requires States to ensure the justiciability of the right to health and access by children and their families to remedies in case of violation through civil, criminal or administrative proceedings. Public Interest Litigation recently brought to the Delhi High Court argued that TB policy violated Constitutional rights to life and health and led to the Court ordering that the government meet with the petitioner with the option of reviving the case if action is not taken [67]. However, effective implementation of the right to health also requires the development of other independent, transparent, and accessible accountability mechanisms such as democratically elected local health councils, patients’ committees, health commissioners, and national human rights institutions (which sometimes have quasi-judicial functions as well) to facilitate access to information, participation and access to remedies. Furthermore, CRC General Comment 15 emphasizes that “States parties to the Convention have obligations not only to implement children’s right to health within their own jurisdiction, but also to contribute to global implementation through international cooperation”, echoing the Committee on Economic, Social and Cultural Rights’ General Comment No. 14 on the right to health, which adds that States Parties should avail themselves of technical assistance available from WHO, UNICEF and others [31, 68].

The right to health also goes beyond legal remedies to guarantee effective delivery of health services, utilizing the “Availability, Accessibility, Acceptability, and Quality” (AAAQ) framework, which is derived from analysis of health service delivery [69]. The AAAQ framework can be used to review paediatric TB services, and in particular to diagnose why children are not receiving sufficient services in each of these dimensions, as a basis for health workers to strategize how to overcome these barriers.

As health practitioners, we can document and report violations of children’s human rights on individual and systems levels, and use the CRC to leverage States parties to deliver personnel, infrastructure and funding for interventions that we already know to be effective: contact tracing, preventive therapy, childhood TB training for health workers, and infection control. As a community, our advocacy strategy needs to be stronger and inclusive, and to give voice to children and families directly affected by TB. Where separation of family members is absolutely necessary, it should be for the minimum possible duration of time, and arrangements made to minimize impact. We need to break free of our health silo and engage with our education and social care colleagues, to highlight the indivisibility and interdependence of children’s rights and the need for multidisciplinary engagement to help children fulfill their potential. In this era of mass migration, Governments must be held to account to provide for all children within their jurisdiction, not merely their own citizens. Fundamentally, the human rights-based approach means the TB together with the child health community needs to join ranks with education and social care agencies to combat poverty and social inequality, which contribute so much to the burden of disease faced by children.

Having described in detail the many instances where children affected by TB are denied their rights, we must now accelerate our efforts to protect the rights of children affected by TB, to reach the most marginalized and excluded, and to change our mindset to use human rights-based approaches to truly leave no child behind.

## Abbreviations

AAAQ: Availability, Accessibility, Acceptability, and Quality
AIDS: Acquired Immune Deficiency Syndrome
CESR: Committee on Economic Social and Cultural Rights
CRC: Convention on the Rights of the Child
HIV: Human Immunodeficiency Virus
HRBA: Human Rights-Based Approach
*M. tuberculosis*: *Mycobacterium tuberculosis*
MDR-TB: Multidrug-resistant tuberculosis (*Mycobacterium. tuberculosis* resistant to two first-line drugs: rifampicin and isoniazid)
TB: Tuberculosis
The Committee: The Committee on the Rights of the Child
UNICEF: United Nations Children’s Fund
WHO: World Health Organization
